# Comparable Efficacy of Oral Bendamustine versus Intravenous Administration in Treating Hematologic Malignancies

**DOI:** 10.21203/rs.3.rs-3848777/v1

**Published:** 2024-01-16

**Authors:** Megan J. Cracchiolo, Lisa Davis, Andrew P. Matiatos, Dan W. Davini, Muhammad Husnain, Richard J. Simpson, Vasilios Voudouris, Emmanuel Katsanis

**Affiliations:** University of Arizona; University of Arizona; University of Arizona; University of Arizona; University of Arizona; University of Arizona; University of Arizona

**Keywords:** Bendamustine, pharmacokinetics, lymphoma, multiple myeloma, acute lymphoblastic leukemia

## Abstract

**Purpose:**

The purpose of this study was to analyze potential differences in antitumor efficacy and pharmacokinetics between intravenous (IV) bendamustine (BEN) and a novel orally administered bendamustine agent (PO) that is utilizing the beneficial properties of superstaturated solid dispersions formulated in nanoparticles.

**Methods:**

Pharmacokinetics of IV versus PO BEN were determined by analysis of plasma samples collected from NSG mice treated with either IV or PO BEN. Plasma samples were analyzed using liquid chromatography-mass spectrometry (LC/MS/MS) following a liquid-liquid extraction to determine peak BEN concentration (Cmax), area under the concentration-time curve (AUC) and the half-life (t1/2) *in-vivo. in-vitro* cytotoxicity of BEN against human non-Hodgkin’s Burkitt’s Lymphoma (Raji), multiple myeloma (MM.1s), and B-cell acute lymphoblastic leukemia (RS4;11) cell lines was determined over time using MTS assays. Luciferase-tagged versions of the aforementioned cell lines were used to determine *in-vivo* BEN cytotoxicity of IV versus PO BEN at two different doses.

**Results:**

Bendamustine at a high dose *in-vitro* causes cell death. There was no significant difference in antitumor efficacy between IV and novel PO BEN at a physiologically relevant concentration in all three xenograft models. *In-vivo* pharmacokinetics showed the oral bioavailability of BEN in mice to be 51.4%.

**Conclusions:**

The novel oral BEN agent tested exhibits good oral bioavailability and systemic exposure for *in-vivo* antitumor efficacy comparable to IV BEN. An oral BEN formulation offers exciting clinical potential as an additional method of administration for bendamustine and warrants further evaluation in clinical studies.

## INTRODUCTION

Bendamustine (BEN) is an intravenously (IV) administered chemotherapeutic agent used primarily against hematologic malignancies. Early testing examined the use of BEN in the context of multiple myeloma as well as breast and small cell lung cancers [1–5]. Later, BEN was found to have activity against chronic lymphocytic leukemia (CLL), Hodgkin’s and non-Hodgkin’s lymphomas (NHL) [2,6–8]. Market availability of the drug began in Germany in the 1970s under the name Cytostasan^®^, and later was re-marketed as Ribomustin^®^ [2,3]. Today, BEN is sold in the United States under the brand names Belrapzo^®^, Bendeka^®^, and Treanda^®^, and is approved for the treatment of CLL and NHL [9].

Nitrogen mustard analogues are synthesized from mustard gas with the replacement of sulfur with nitrogen. Bendamustine (4-(5-[bis(2-chloroethyl)amino]-1-methyl-2-benzimidiazolyl) butyric acid hydrochloride) contains a benzimidazole ring which is thought to confer anti-metabolite properties in addition to the mechlorethamine group and butyric acid side chain [10,2,7,11]. The butyric acid side chain confers hydrophilic properties to BEN, allowing for solubility in water [11]. As an alkylating agent, BEN can create inter- and intra- strand lesions in DNA, leading to cell death in proliferative cells such as cancerous and hematopoietic cells [7,2]. Compared to other alkylating agents such as cyclophosphamide, BEN induces more DNA lesions at equitoxic concentrations and has been shown in early studies to activate a base excision DNA repair pathway, a unique method of action for an alkylating agent [3,2,8]. While specific mechanisms are still to be determined, BEN has been shown to induce apoptosis through a p53-dependant pathway, and exposure to BEN *in-vitro* can lead to mitotic catastrophe whereby cells are inhibited at several mitotic checkpoints with severe DNA damage and subsequent cell death [3,2,8].

BEN is considered safe and well tolerated, with adverse effects including neutropenia, lymphopenia, nausea, and vomiting [6]. We have studied the immunomodulatory properties of BEN as a chemotherapeutic in the contexts of pre- allogeneic hematopoietic cell transplant (HCT) conditioning and as a post-transplant graft-versus-host-disease chemotherapeutic, targeting alloreactive T-cells [12–19]. Recently, BEN has been used to replace fludarabine and cyclophosphamide combination regimens for lymphodepletion in the context of pre-CAR therapies [20–22].

In humans, BEN is typically given on two consecutive days, intravenously over 30 to 60 minutes. Pharmacokinetic data in humans have shown the effective half-life to be about forty minutes [11]. The current parenteral administration of BEN requires treatment to take place in the clinic or hospital. The presence of an effective and pharmacologically comparable oral formulation of BEN would have advantages for the patient and the clinic. An oral (PO) form of BEN would allow for patients to receive treatment at home reducing travel, time commitment and financial burden on patients and caretakers. Previously, Shimizu et al. demonstrated that BEN given through liquid-filled hard capsules was a safe method of delivery with mild gastrointestinal distress in patients with advanced solid tumors [23]. Herein we investigated the pharmacokinetics and anti-tumor effects of a novel, PO form of BEN that is a supersaturated solid dispersion formulated in nanoparticles and is administered by oral gavage to immunodeficient NSG mice bearing human hematologic cancers and compared its efficacy and toxicity to commercially available IV BEN.

## MATERIALS & METHODS

### Mice

NSG (NOD.Cg-Prkdc^scid^ Il2rg^tm1Wjl^/SzJ) mice were purchased from The Jackson Laboratory and bred at the University of Arizona Experimental Mouse Shared Resource. Female mice ages 6–14 weeks were randomized by weight and used for experiments. All mice had ad libitum access to food and water and were maintained on a twelve-hour light-dark cycle in specific-pathogen free conditions. The University of Arizona Institutional Animal Care and Use Committee (IACUC) approved all protocols and plans for the experiments.

### Mass Spectrometry and Pharmacokinetics

Sixteen NSG mice were allocated to two groups (8 mice per group) and administered a single dose of Bendamustine (BEN) either intravenously (IV) or by oral gavage (PO). BEN for IV administration (SelleckChem no. S1212) was solubilized in DMSO, diluted with PBS containing 0.2% carboxymethylcellulose and 0.25% polysorbate 80 and dosed at 15mg/kg. PO BEN was obtained from Exinda Therapeutics and sealed with desiccant in amber glass containers to avoid exposure to air or moisture. To prepare PO BEN (17.65 mg/kg), the appropriate amount of drug was weighed in a sterile hood and solubilized with filtered water. DMSO was added to solubilized PO BEN aliquots at a 5% concentration to control for the difference in the IV versus PO preparations. No precipitation was observed post solubilization. PO BEN was administered to mice by oral gavage within one hour of preparation. IV BEN was administered to mice via tail vein injection. At 0.25, 0.5, 1, 2, 4, and 6 hours after BEN administration, mice (4 mice per sampling time point) were bled via tail bleed into EDTA tubes. Peripheral blood was centrifuged, and plasma collected and stored at −80 degrees. Plasma samples were transferred to the University of Arizona Cancer Center Analytical Chemistry Shared Resource for analysis via liquid chromatography-mass spectrometry (LC/MS/MS) using a modification of previously validated methods [24,25]. The average plasma concentration versus time data were analyzed using PKSolver 2.0 using a noncompartmental approach.

### Cell Culture

Raji (ATCC, CCL-86, 70053718), MM.1s (ATCC, CRL-2974, 70042525), and RS4;11 (ATCC, CRL-1873, 70036117) were thawed from liquid nitrogen and cultured in RPMI-1640 (Cytiva, SH30027.01) with 10% FBS, HEPES (Gibco, 15630–080), Sodium Pyruvate (Cytiva, SH30239.01), and Penicillin/Streptomycin (P/S, Gibco, 15140–122). Cells were kept at 37°C and 5% CO_2_. Cells were maintained by the addition of media every one to three days.

### MTS Assays

Raji (50,000 cells/well), MM.1s (40,000 cells/well), or RS4;11(40,000 cells/well) were plated in a 96-well plate in 100uL total volume. BEN (75mg/mL stock) was diluted in media and added in a serial dilution in triplicate. Control wells containing media only and media with BEN were plated for control. Plates were kept in an incubator at 37°C and 5% CO_2_ for 24 to 72 hours. The plates were read at 490 nm (PowerWave XS, BioTek) four hours after 20uL of reagent was added to each well using the CellTiter 96 Aqueous One Solution Cell Proliferation Assay (VWR, Promega Corporation, Madison, WI, USA). Data was read using BioTek Gen 5 Microplate Software. Background absorbance from the media-only or media and drug wells was subtracted from each corresponding concentration data point. The percent viability was normalized to the cell-only control. Data was averaged from the three triplicate values per each plate. Each experimental plate was repeated three times in total. Data was inputted and graphed using GraphPad (Prism).

### Tumor Models

Raji-Luciferase (Raji-Luc, ATCC, CCL-86-LUC2, 70059299), MM. 1s-Luc (Fenics Bio, CL-1617, 080721), and RS4;11-Luc (Fenics Bio, CL-1221,) were thawed from liquid nitrogen and maintained in RPMI-1640 with 10% FBS, HEPES, Sodium Pyruvate, and P/S. Following three washes in 1xPBS, cells were administered to mice via tail vein injection in 100uL total volume 24 hours following a 150cGy total body irradiation with a RadSource X-ray irradiator. For the Raji-Luc or MM.1s-Luc model, 0.2×10^6^ cells were given one day after thaw. For RS4;11-Luc, 1×10^6^ cells were administered after a six day expansion culture. Cells were spun down and washed in 1xPBS (Cytiva, SH30256.01) three times before being resuspended in the aforementioned concentrations. NSG mice were given either IV BEN (15mg/kg or 30mg/kg) or PO BEN (30mg/kg or 60mg/kg) on days 1 and 2 for the MM.1s-Luc and RS4;11-Luc models and on days 3 and 4 for the Raji-Luc model. The dose difference between IV and PO is due to bioavailability adjustment to maintain equivalence. Mice were monitored daily for morbidity and weighed two to three times weekly. Animals losing more than thirty percent of starting weight for two consecutive weight scores or those experiencing total hind-limb paralysis were sacrificed.

### Bioluminescence imaging

Two to three times weekly, mice received a 200uL intraperitoneal injection of D-luciferin (15mg/mL, Gold Bio), were anesthetized with 2% isoflurane, and imaged using the Spectral Instruments LagoX (Tucson, AZ, USA) system five to ten minutes later. Images were taken over a 5-minute exposure time. For visual representative images, scales were adjusted to a radiance scale minimum of 1.7×10^4^ and maximum of 5.5×10^7^ and saved as JPEG files. Bioluminescence was quantified using the Aura imaging software (Spectral Instruments Imaging, Tucson, AZ, USA) and are presented as photons/second in a region of interest that included the entire animal. Data was inputted and graphed using GraphPad (Prism).

### Statistics

GraphPad Prism 9 (La Jolla, CA) was used for statistical analysis. IC50 values were determined for each cell line at each time point using a nonlinear fit [Inhibitor] vs. response – variable slope (four parameter). Survival Kaplan-Meir curves were analyzed using a Log-rank Mantel-Cox test[26,27]. P-values of <0.05 were considered significant, and the asterisks indicate increasing levels of significance with *<0.05, ** <0.01, ***<0.001, ****<0.0001.

## RESULTS

### The novel PO BEN exhibits good oral bioavailability and achieves clinically relevant systemic exposure.

**Figure 1:**
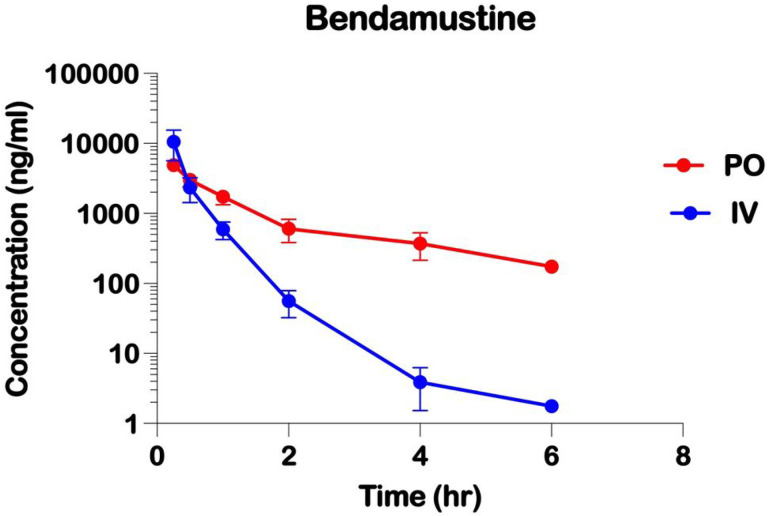
Pharmacokinetics of Intravenous and Oral Administrations of Bendamustine in NSG mice. Plasma concentrations of Bendamustine delivered either (IV) by tail vein injection or (PO) orally by gavage. Plasma concentrations of Bendamustine were determined by liquid chromatography-mass spectrometry (LC/MS) following liquid-liquid extraction.

The plasma concentration versus time profiles with IV and PO BEN are shown (Figure 1). The highest plasma BEN concentrations were observed at the first sample time of 0.25 hours, indicating rapid oral absorption. Plasma concentrations declined slowly as a function of time with an effective terminal half-life of 0.53 and 2.22 hours, following IV and PO doses, respectively. A PO BEN dose of 17.65 mg/kg produced an average maximum plasma concentration (Cmax) of 4872.1 ng/mL compared to an average Cmax with IV BEN dose of 15 mg/kg of 10518.4 ng/mL. The area under the plasma concentration versus time curve from time 0 extrapolated to infinity (AUC_0−∞_) was 9973.3 ng h/mL with IV BEN compared to 6031.9 ng h/mL with PO BEN, indicating an oral bioavailability of 0.514.

### Bendamustine has a dose dependent but variable cytotoxicity against hematologic cancer cell lines in-vitro.

**Figure 2:**
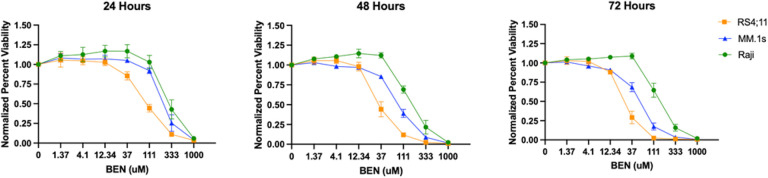
In vitro cytotoxicity of Bendamustine against human hematologic malignancy cell lines. Dose response curves for RS4;11, MM.1s, and Raji. Tumor lines were plated in triplicate with various concentrations of Bendamustine (uM) as indicated for (a) 24, (b) 48, or (c) 72 hours. Plates were read using the CellTiter 96 AQueous One Solution Cell Proliferation (MTS) Assay and read at 490nm on a plate reader 4 hours after administration of reagent to each well. Normalized percent viability for three averaged experiments is shown at each BEN concentration.

After 24, 48, and 72 hours of exposure to BEN *in-vitro*, MTS data showed sensitivity of RS4;11, MM.1s, and Raji cell lines to BEN induced cell death. RS4;11, a B-cell acute lymphoblastic leukemia, showed decreased cell viability as compared to Raji, a Non-Hodgkin’s Burkitt’s Lymphoma, and MM.1s, a multiple myeloma, for all time points (Figure 2). At 24 hours, RS4;11 had the lowest IC50 (80 uM) while MM.1s and Raji had IC50s of 210 uM and 270 uM, respectively. At 48 hours, the IC50 concentrations were 32 uM (RS4;11), 87 (MM.1s), and 143 (Raji). Finally, at 72-hours, all cell lines exposed to BEN resulted in the lowest IC50 values across the three time points. RS4;11, MM.1s, and Raji had 72-h IC50 values of 25 uM, 54 uM, and 133 uM respectively. All cell lines maintained some resistance to BEN-induced killing at low concentrations below 12.34 uM.

### RS4;11 B-cell acute lymphoblastic leukemia (B-ALL) xenograft model.

**Figure 4:**
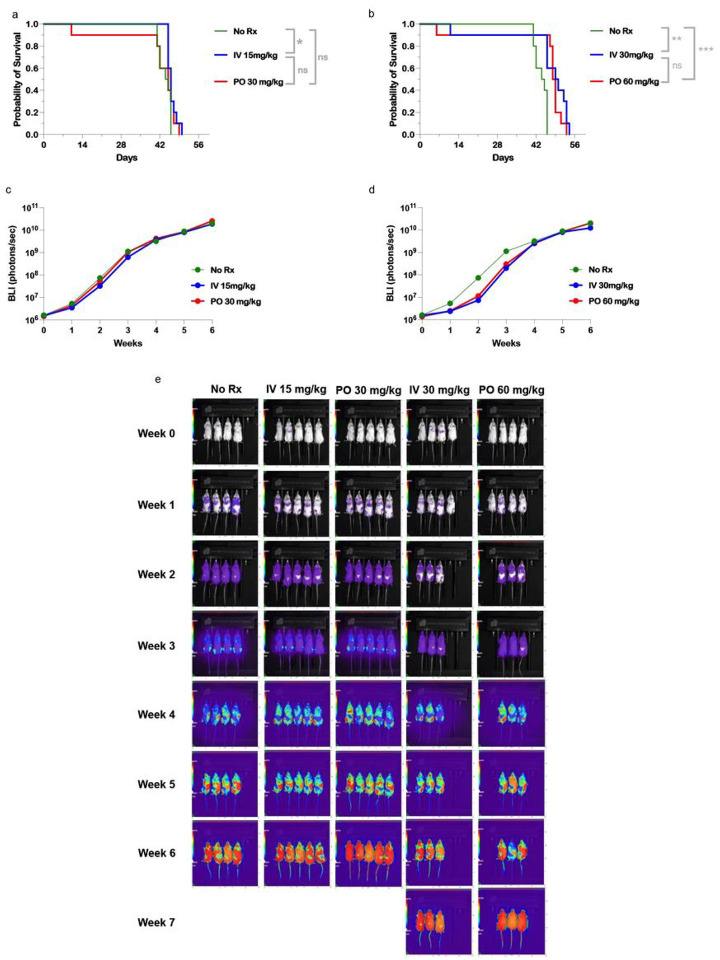
In vivo application of IV versus PO Bendamustine treatment in a B-Cell Acute Lymphoblastic Leukemia xenograft model. NSG mice received 150cGy TBI on Day −2 and were injected on Day −1 with 1×10^6^ luciferase-tagged RS4;11 cells by tail vein injection. Bendamustine was administered on Days 3 and 4 either intravenously by tail vein injection (IV, 15 mg/kg or 30 mg/kg) or by oral gavage (PO, 30mg/kg or 60 mg/kg). (a,b) Kaplan-Meier curves shows survival of NSG mice with comparable doses of IV or PO BEN. Mice were sacrificed if they reached two consecutive weight loss scores greater than thirty percent of starting weight or if moribund. (c,d,e) Bioluminescence imaging was conducted two to three times weekly and (c,d) photons/second in a region of interest encompassing the entire animal are shown as average over time, n= 10 per group. (e) Representative images from one experiment are shown with the same rainbow coloring set to the same min and max radiance. Experiments were repeated twice. A log-rank (Mantel-Cox) test was performed for survival analysis. P-values of <0.05 were considered significant, and the asterisks indicate increasing levels of significance with *<0.05, **<0.01, ***<0.001, ****<0.0001.

In the mouse xenograft model of a B-cell acute lymphoblastic leukemia, RS4:11, there was no difference between IV and PO BEN at both the lower doses (Fig 4a, IV 15 mg/kg or PO 30 mg/kg) or higher doses (Fig 4b, IV 30 mg/kg or PO BEN 60 mg/kg). Surprisingly, given its increased sensitivity to BEN *in-vitro* only the higher doses of IV and PO BEN impacted survival. Using IV 30mg/kg and PO 60 mg/kg, both treatments resulted in a modest yet significantly improved survival over the control group. All treatment groups showed a modest decrease in body weight following BEN dosing, with a recovery to near starting weight, and there was no difference in appearance of the mice between the groups. (Data not shown). Additionally, bioluminescence imaging quantified to photons/second shows no difference between the low doses and higher doses (Fig 4c, IV 15 mg/kg or PO 30 mg/kg) and lower doses of BEN (Fig 4d, IV 15 mg/kg or PO 30 mg/kg).

### MM. 1s multiple myeloma xenograft model

**Figure 5:**
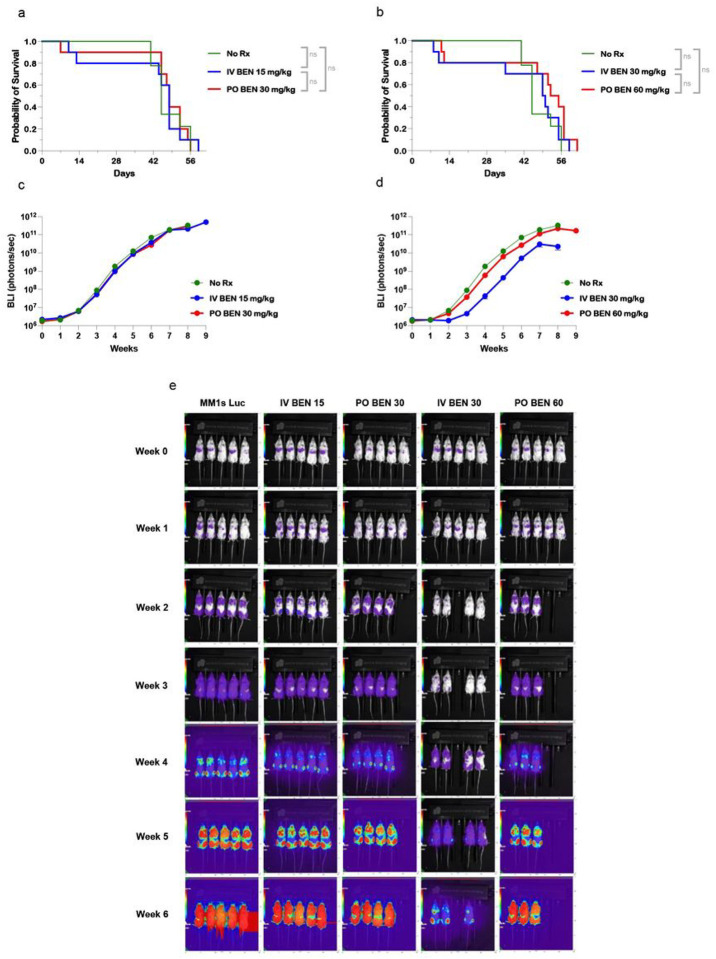
In vivo application of IV versus PO Bendamustine treatment in a Multiple Myeloma xenograft model. NSG mice received 150cGy TBI on Day −2 and were injected on Day −1 with 2×10^5^ luciferase-tagged MM.1s cells by tail vein injection. Bendamustine was administered on Days 1 and 2 either intravenously by tail vein injection (IV, 15 mg/kg or 30 mg/kg) or by oral gavage (PO, 30mg/kg or 60 mg/kg). (a,b) Kaplan-Meier curves shows survival of NSG mice with equivalent doses of IV or PO BEN. Mice were sacrificed if they reached two consecutive weight loss scores greater than thirty percent of starting weight or if hind limb paralysis was reached. (c,d,e) Bioluminescence imaging was conducted two to three times weekly and (c,d) photons/second are shown as average over time, n= 9–10 per group. (e) Representative images are shown according to the same rainbow coloring set to the same min and max radiance. Experiments were repeated twice. A log-rank (Mantel-Cox) test was performed for survival analysis. P-values of <0.05 were considered significant, and the asterisks indicate increasing levels of significance with *<0.05, ** <0.01, ***<0.001, ****<0.0001.

The xenograft multiple myeloma model using MM.1s-Luciferase cells demonstrated no survival difference between control (No Rx) and any treatment group (Fig 5a,b). Despite no significance in survival, the high dose (30 mg/kg) of IV BEN showed a modest trend toward decreased bioluminescence imaging signal (Fig 5d). There was no visual difference between the mice in any group, and weight was comparable between treatment groups with weight loss seen in conjunction with development of tumor for all groups (Data not shown).

### Raji Burkitt’s lymphoma xenograft model

**Figure 6:**
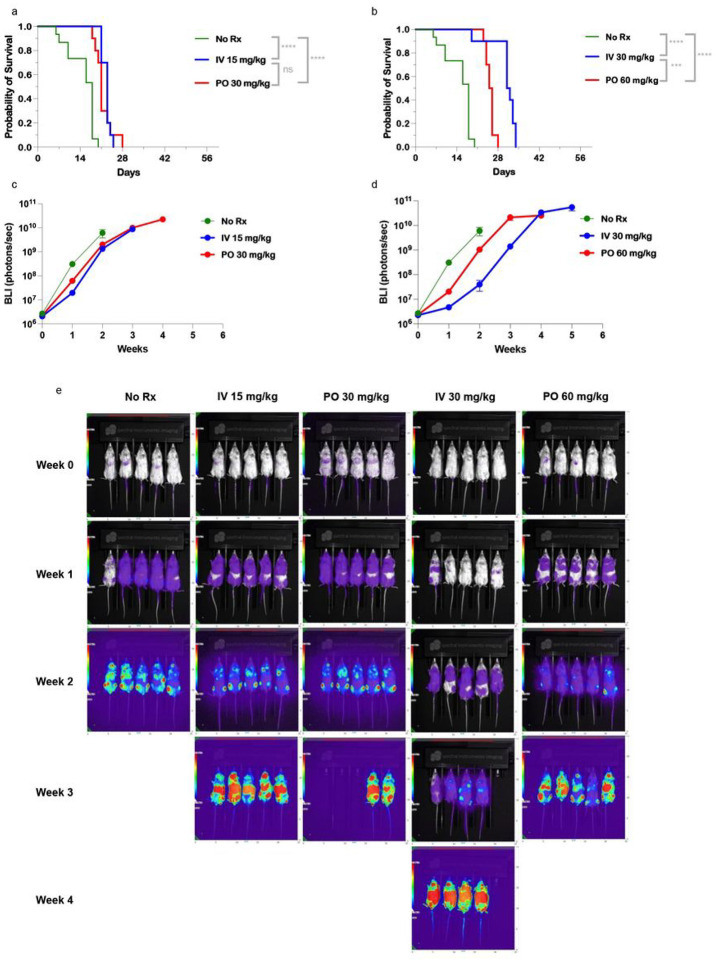
In vivo application of IV versus PO Bendamustine treatment in a non-Hodgkin’s Burkitt’s Lymphoma xenograft model. NSG mice received 150cGy TBI on Day −2 and were injected on Day −1 with 2×10^5^ luciferase tagged Raji cells by tail vein injection. Bendamustine was administered on Days 1 and 2 either intravenously by tail vein injection (IV)(15 mg/kg or 30 mg/kg) or by oral gavage (PO)(30mg/kg or 60 mg/kg). (a,b) Kaplan-Meier curves shows survival of NSG mice with equivalent doses of IV or PO BEN. Mice were sacrificed if they reached two consecutive weight loss scores greater than thirty percent of starting weight or if hind limb paralysis was reached. (c,d,e) Bioluminescence imaging was conducted two to three times weekly and (c,d) photons/second are shown as average over time, n= 10–15 per group. (e) Representative images are shown according to the same rainbow coloring set to the same min and max radiance. Experiments were repeated twice. A log-rank (Mantel-Cox) test was performed for survival analysis. P-values of <0.05 were considered significant, and the asterisks indicate increasing levels of significance with *<0.05, **<0.01, ***<0.001, ****<0.0001.

Using Raji, a Burkitt’s lymphoma, NSG mouse model we found no significant difference between pharmacokinetically comparable lower doses of IV vs PO BEN (Fig 6a, 15 mg/kg or 30mg/kg, respectively) however, both treatment groups had significantly extended survival as compared to the control (IV 15 mg/kg vs. No Rx; PO 30 mg/kg vs. No Rx). In this Burkitt’s lymphoma, which was the most resistant *in-vitro* (Figure 2), doubling the dose of both IV and PO BEN conferred prolonged survival, with greater improvement seen with IV over PO, leading to a significant difference between them (Fig 6b). Both at the lower doses and higher doses, modest weight loss was seen in the treatment groups, however there was no difference in appearance of the mice between groups (Data not shown). For bioluminescent imaging, IV BEN at 30 mg/kg showed a decreased tumor burden at week 3 compared to other treatment groups (Fig 6d,e). However, mice only survived approximately one more week when compared to PO BEN.

## DISCUSSION

We have successfully shown that a newly developed orally bioavailable formulation of Bendamustine (BEN), when appropriately dosed and administered, yields comparable cytotoxic effects to BEN administered intravenously (IV) against hematologic malignancies in our mouse models for lymphoma, multiple myeloma, and leukemia. As BEN is currently administered exclusively through IV routes in clinical practice, patients are constrained to in-hospital treatments. Therefore, the availability of an effective oral form of BEN is essential to enable the expansion of out-of-hospital treatment options for patients. It would further reduce the overall cost of treatment for patients and healthcare systems and enhance treatment compliance especially on certain classes of older and frail patients.

While our pharmacokinetic data in mice is promising, analysis of the novel oral BEN pharmacokinetics must be examined in humans to determine safety and efficacy in adult and pediatric patients. Comparing IV to PO doses of BEN (15 mg/kg IV and 17.65 mg/kg PO, respectively) the Cmax values of 10518.4 ng/mL and 4872.1 ng/mL correspond to micromolar (uM) concentrations of 29uM and 14 uM respectively. Previous pharmacokinetic data of BEN in humans reported peak plasma concentrations of 0.1–0.3 ug/mL for doses of 30 to 200 mg/m^2^, and an effective half-life of around 40 minutes [11,28]. With our IV BEN pharmacokinetic data showing a half-life of 0.53 hours, the IV duration of the drug is similar. However, our PO BEN showed a half-life of 2.22 hours, with a maximum plasma concentration that is consistent with a bioavailability of 51.4%. Although the longer half-life observed with PO BEN was longer compared to that with IV BEN, it likely represents continued absorption from the gastrointestinal tract. Additional studies are needed to better describe the systemic exposure and terminal half-life with PO BEN with extending sampling times and multiple dose levels.

Our *in-vitro* data of BEN’s cytotoxic effects against three hematological malignancy cell lines demonstrated surprising results. BEN is a chemotherapeutic agent typically given for chronic lymphocytic leukemia, indolent B-cell non-Hodgkin lymphoma, and multiple myeloma. We demonstrated here that across varying concentrations of BEN, RS4;11, a B-cell acute lymphoblastic leukemia, was most sensitive to BEN-induced killing *in-vitro*. However, our *in-vivo* data demonstrated that only some experimental groups had a minimal but statistically significantly increased survival compared to untreated controls. MM.1s, a multiple myeloma cell line, displayed intermediate sensitivity in our *in-vitro* testing. This outcome is in line with expectations, given the established efficacy of BEN against multiple myeloma, both as a standalone treatment and as part of combination therapy, particularly in cases of relapsed or refractory multiple myeloma [29,30]. In line with our data, previous studies have reported MM.1s sensitivity to BEN *in-vitro* at concentrations higher than 100 umol/L at 72 hours [31]. In our mouse xenograft model, however, there was no survival advantage for mice receiving BEN as compared to No Rx. A slight decrease in bioluminescence signal at IV BEN 30 mg/kg demonstrates that high doses of BEN may decrease early tumor burden in NSG mice. However, the MM.1s-Luciferase model resulted in saturating signal (photons/second) for every group at our given dose of tumor cells. Lastly, the most resistant cell line *in-vitro* was Raji, a non-Hodgkin’s Burkitt’s lymphoma. Despite resistance in our MTS assays, BEN *in-vivo* prolonged survival of mice in all treatment groups as compared to the controls. No cell line in the xenograft mouse models demonstrated complete clearance of tumor, offering a window for possibilities of studying the immunomodulatory effects of BEN with immunocompetent mice, or NSG mice engrafted with human immune cells.

Across our models, there were limited differences between IV and PO formulations of BEN, especially at the lower doses of 15 mg/kg and 30mg/kg respectively. After examining the pharmacokinetic data and comparing the area under the curve (AUC inf, ng/mL*h), the oral bioavailability was determined to be 514 of the IV dose. Using this value, equivalent PO doses were given as double that of IV, with the assumption that the oral bioavailability data is similar at higher doses of PO BEN (30 mg/kg and 60 mg/kg). Differences were seen between IV and PO BEN in the Raji-Luc model when doses of BEN were doubled to 30 mg/kg and 60 mg/kg for IV and PO BEN respectively. While we have yet to elucidate why these differences exist, one can speculate that differences in peak plasma concentration or differences in absorption time profiles may play a role in IV extending survival against the tumor. Additionally, there are clear differences between the biological processing of the drugs. IV administration bypasses the need for gastrointestinal absorption of the drug. Since there is limited data on the pharmacokinetic mechanisms of BEN, it is also possible that IV administration bypasses proteins or membrane transporters that can alter intracellular exposure as BEN has been demonstrated to be a substrate for P-glycoprotein and breast cancer resistance protein (BCRP)[9].

The availability of oral BEN has the potential to significantly enhance accessibility and reshape the options for dosing schedules for patients. Administering the medication orally enables the possibility of safe and effective therapy that is more convenient and uses fewer resources, opening new avenues for novel treatment regimens. The utilization of oral BEN warrants human trials to assess its safety, validate its pharmacokinetics, and investigate any potential nutritional or drug interactions that may arise due to this alternative delivery method.

## Figures and Tables

**Figure 3:**
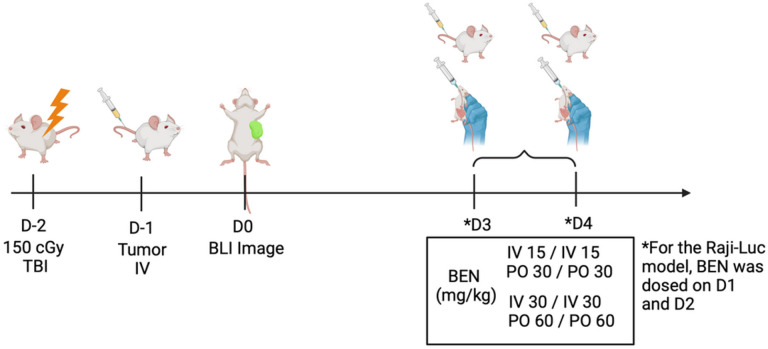
Schematic diagram of experimental layout. NSG mice received 150cGy TBI on Day −2 and were injected on Day −1 with tumor cells by tail vein injection. Bendamustine was administered on Days 3 and 4 either intravenously by tail vein injection (IV, 15 mg/kg or 30 mg/kg) or by oral gavage (PO, 30mg/kg or 60 mg/kg) for the MM.1s-Luc and RS4;11-Luc models. For the Raji-Luc model, BEN was administered on Days 1 and 2. Image created with BioRender.com.

## Data Availability

The authors do not have research data declarations to make.
